# Low-dose CT imaging of a total hip arthroplasty phantom using model-based iterative reconstruction and orthopedic metal artifact reduction

**DOI:** 10.1007/s00256-017-2580-2

**Published:** 2017-02-15

**Authors:** R. H. H. Wellenberg, M. F. Boomsma, J. A. C. van Osch, A. Vlassenbroek, J. Milles, M. A. Edens, G. J. Streekstra, C. H. Slump, M. Maas

**Affiliations:** 1 Department of Radiology, Academic Medical Center, Meibergdreef 9, 1105 AZ Amsterdam, The Netherlands; 2 Department of Radiology, Isala, Zwolle, The Netherlands; 3 Philips Medical Systems, Brussels, Belgium; 4 Philips Medical Systems, Eindhoven, The Netherlands; 5 Department of Innovation and Science, Isala, Zwolle, The Netherlands; 6 MIRA Institute for Biomedical Technology and Technical Medicine, University of Twente, Enschede, The Netherlands

**Keywords:** Computed tomography, Metal artifacts, Radiation dose reduction, Model-based iterative reconstruction, IMR, Total hip arthroplasty phantom, Quantitative analysis, O-MAR

## Abstract

**Objective:**

To compare quantitative measures of image quality, in terms of CT number accuracy, noise, signal-to-noise-ratios (SNRs), and contrast-to-noise ratios (CNRs), at different dose levels with filtered-back-projection (FBP), iterative reconstruction (IR), and model-based iterative reconstruction (MBIR) alone and in combination with orthopedic metal artifact reduction (O-MAR) in a total hip arthroplasty (THA) phantom.

**Materials and methods:**

Scans were acquired from high- to low-dose (CTDI_vol_: 40.0, 32.0, 24.0, 16.0, 8.0, and 4.0 mGy) at 120- and 140- kVp. Images were reconstructed using FBP, IR (iDose^4^ level 2, 4, and 6) and MBIR (IMR, level 1, 2, and 3) with and without O-MAR. CT number accuracy in Hounsfield Units (HU), noise or standard deviation, SNRs, and CNRs were analyzed.

**Results:**

The IMR technique showed lower noise levels (*p* < 0.01), higher SNRs (*p* < 0.001) and CNRs (*p* < 0.001) compared with FBP and iDose^4^ in all acquisitions from high- to low-dose with constant CT numbers. O-MAR reduced noise (*p* < 0.01) and improved SNRs (*p* < 0.01) and CNRs (*p* < 0.001) while improving CT number accuracy only at a low dose. At the low dose of 4.0 mGy, IMR level 1, 2, and 3 showed 83%, 89%, and 95% lower noise values, a factor 6.0, 9.2, and 17.9 higher SNRs, and 5.7, 8.8, and 18.2 higher CNRs compared with FBP respectively.

**Conclusions:**

Based on quantitative analysis of CT number accuracy, noise values, SNRs, and CNRs, we conclude that the combined use of IMR and O-MAR enables a reduction in radiation dose of 83% compared with FBP and iDose^4^ in the CT imaging of a THA phantom.

## Introduction

Computed tomography (CT) is an imaging modality widely used for postoperative follow-up in patients after total hip arthroplasty (THA). The CT imaging of metal hip prosthesis results in metal artifacts due to photon-starvation, beam-hardening, and scatter [1], which impede the detection of prosthetic-related pathological conditions of soft tissues and bone. the soft tissue and bone.

The orthopedic metal artifact reduction algorithm, O-MAR, is an iterative metal artifact reduction algorithm specially developed for CT imaging of large metal orthopedic implants [2]. O-MAR is a sinogram inpainting technique that identifies and replaces those projections that passed through metal with interpolated data from adjacent projections that did not pass through metal. With O-MAR, Hounsfield Units (HUs) are corrected toward baseline levels and contrast-to-noise-ratios (CNRs) are boosted [3–6]. Recently, we showed in a phantom study that O-MAR significantly reduces metal artifacts when combined with iDose^4^ and IMR, which are Philips’ proprietary iterative reconstruction (IR) technique and model-based iterative reconstruction technique (MBIR) respectively [7, 8]. CT number accuracy, signal-to-noise-ratios (SNRs), and CNRs were significantly improved, whereas noise values decreased. We found that IMR strongly improves overall image quality and that O-MAR is most effective in reducing severe metal artifacts and when combined with IMR compared with iDose^4^ and filtered back-projection (FBP) using a large head metal-on-metal (MoM) THA phantom [8]. O-MAR post-processes the projection data, taking into account metal-only classified images, tissue-classified images, and original input images, thereby providing more regular attenuation profiles before image reconstruction, which can improve the general performance of iDose^4^ and IMR [2].

Besides improved overall image quality using MBIR techniques such as IMR at similar radiation dose levels, the use of IMR is expected to allow a radiation dose reduction [9–16]. The rationale behind this assumption is that model-based iterative reconstruction techniques are more capable of handling increased detector noise levels at a reduced dose compared with the standard reconstruction technique, FBP, and iterative reconstruction techniques as it incorporates data statistics, image statistics, and system models. Furthermore, IMR does not involve blending with FBP such as hybrid iterative reconstruction techniques, which results in significantly better image quality. Using low-dose protocols, while maintaining image quality, could increase the acceptance of using CT in the clinical routine of orthopedic imaging owing to the reduction of radiation exposure to the orthopedic patient population. To test the hypothesis that it is possible to lower the radiation dose while maintaining sufficient image quality, or even improving image quality, in a challenging population, we performed this phantom study.

The aim of this study was to compare quantitative measures of image quality, in terms of CT number accuracy, noise, and SNR and CNR values, at different dose levels with FBP, iDose^4^, and IMR alone and in combination with O-MAR in a THA phantom.

## Materials and methods

A THA phantom was scanned on an iCT Brilliance 256-slice CT scanner (Philips Healthcare). Static scan parameters were 64 × 0.625 mm collimation, 0.9-mm slice thickness with 0.45-mm increment, 330 mm field-of-view, 0.398 pitch, 512 × 512 image matrix ,and a rotation time of 1.0 s. The computed tomography dose volume indexes (CTDI_vol_) of a CT scan of the THA phantom while using the CT protocol at 140 kVp is approximately 24.0 mGy using the iterative reconstruction technique iDose^4^ level 4. Scans were acquired from high- to low-dose with fixed CTDI_vol_ of 40.0 (high), 32.0, 24.0, 16.0, 8.0, and 4.0 mGy (low) at 120- and 140- kVp. The higher CTDI_vol_ of 40.0 and 32.0 mGy were taken into account as we were also interested in radiation dose levels in the case of non-iterative reconstruction techniques using FBP. All scans were reconstructed with FBP, iDose^4^ and IMR with and without O-MAR (Philips Healthcare). iDose^4^ can be used at seven different levels of noise reduction where levels 2, 4, and 6 were chosen. For IMR reconstructions, an IMR prototype reconstruction system (version R11) was used. IMR can be used at three levels of noise reduction, which were all investigated. Hard or sharp filter types, which are standard filters for imaging bone structures, were used for all reconstruction methods to increase the contrast-enhanced edges among bone, soft tissues, and prosthesis.

**Fig. 1:**
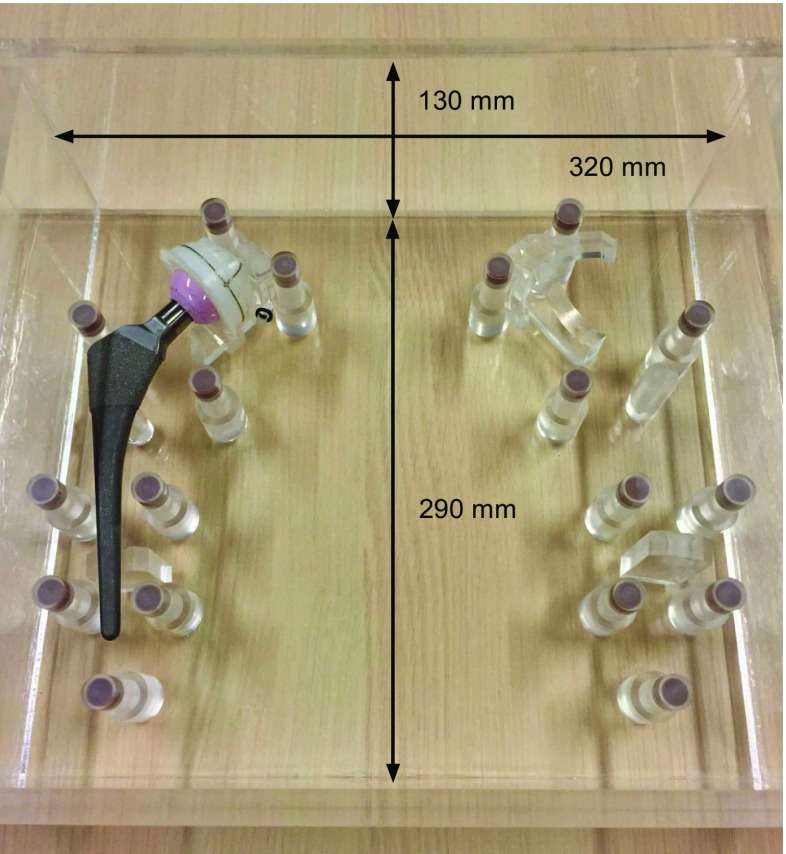
A total hip arthroplasty phantom was used made of polymethyl methacrylate (PMMA), containing a commonly used total hip prosthesis surrounded by 18 hydroxyapatite/calcium carbonate pellets representing bone

The custom-made water-filled THA phantom was made of polymethyl methacrylate (PMMA) with dimensions of 320 mm in width, 130 mm in height, and 290 mm in depth. Additional PMMA shields were placed below and on top of the phantom to increase the sagittal diameter to 190 mm to represent more realistic patient dimensions, based on the water-equivalent diameter (WED) of 29.15 cm and coronal diameter of 320 mm derived from a BMI of 25 using a formula of [17] (Fig. 1). A commonly used total hip prosthesis configuration at our institute was used. The stem consists of a titanium–aluminum–vanadium (Ti_6_Al_4_V) alloy where the head of the prosthesis consists of a zirconia-hardened alumina ceramic. The composition includes SrO, Y_2_O_3_, and Cr_2_O_3_ [18]. The cup is made of ultra-high-molecular-weight polyethylene [19]. The prosthesis was fixated with custom-made PMMA molds to prevent movement. The phantom contains 18 cylindrical hydroxyapatite/calcium carbonate pellets representing bone with a height and diameter of 10 mm. The density of the pellets is calibrated with a documented tolerance of ± 0.5%, simulating healthy bone [7]. On each side 9 pellets were fixated at clinically relevant Gruen zones and DeLee and Charnley zones [20, 21].

**Fig. 2:**
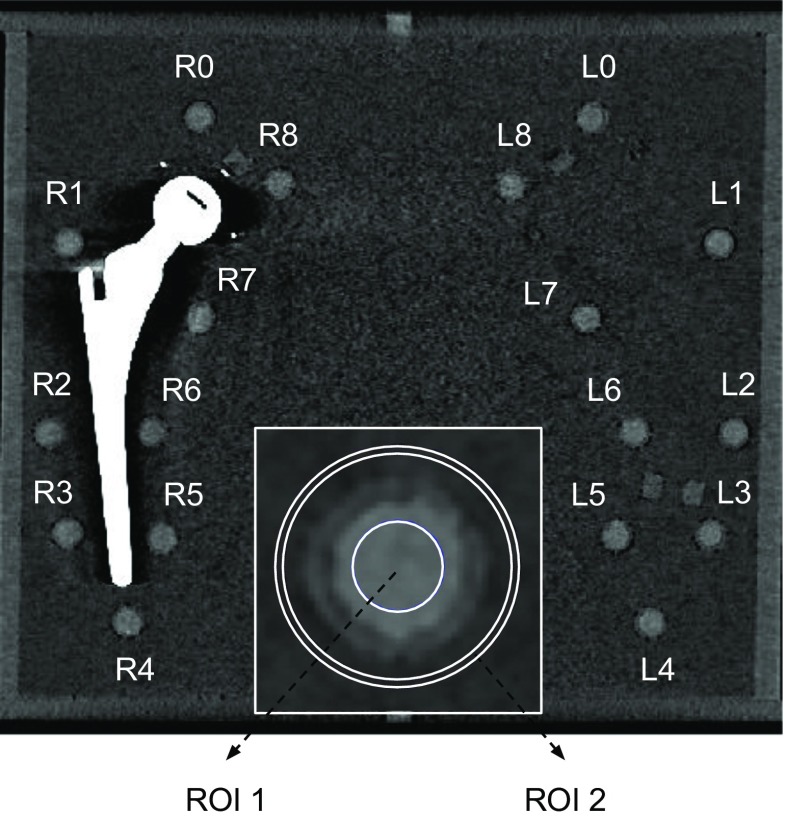
The measurement template mask including the regions of interest (ROIs) of the 18 pellets, 9 left pellets (L0–L8) and 9 right pellets (R0–R8) is shown. A single pellet is enlarged with the inner pellet, ROI 1, and the outer background ROI 2

The effects of radiation dose reduction on overall image quality, metal artifacts, and metal artifact reduction were quantified by analyzing CT numbers in HU, noise levels, SNRs, and CNRs within fixed regions of interest (ROIs). Noise was measured by calculating the standard deviation (SD) of CT values in an ROI. Local SNRs were calculated by dividing CT numbers of the pellet ROI in HU by the standard deviation of the background ROI placed in water. Local CNRs were calculated by subtracting the average HUs of the local background from the average HUs of the pellet and dividing this by the standard deviation of the local background ROI. Coronal DICOM slices, aligned at the middle of the pellets and prosthesis, were used for quantitative measurements. A standardized measurement template was manually created using ImageJ (V 1.48) and consisted of 9 left pellet ROIs (L0–L8) and 9 right pellet ROIs (R0–R8. To enhance the reliability, the measurements were executed using Matlab (version 2014b, Natick, Massachusetts, USA) (Fig. 2). ROIs placed in the pellets had a diameter of 14.7 pixels or 6.6 mm, of the actual 10 mm diameter of the pellet, thus minimizing partial volume effects. The numbers of pixels for the background ROIs and pellet ROIs were matched (Fig. 2).

Pellets L0, L4, R0, and R4 were unaffected by metal artifacts because of their position in the phantom and thereby served as a reference (Fig. 2). The lack of metal artifacts in these four pellets was in concordance with previous work [7, 8]. Reference values regarding CT number accuracy, noise values, SNRs, and CNRs were determined by averaging values of these unaffected pellets, L0, L4, R0, and R4, for each CTDI_vol_ from high-dose (40.0 mGy) to low-dose (4.0 mGy), for 120- and 140-kVp acquisitions, and for each of the reconstructions. In the case of metal artifacts, image quality, metal artifact, and metal artifact reduction were quantified by analyzing CT numbers, noise values, SNRs, and CNRs of the most affected pellet, pellet R6, from high- to low-dose in 120- and 140-kVp acquisitions and these results were compared with reference values of unaffected pellets.

Statistical analysis was performed by means of repeated measures ANOVA (full factorial, type III). For reference values of unaffected pellets one within-subject factor, i.e., reconstruction technique (FBP, iDose^4^ level 2, 4, and 6, and IMR level 1, 2, and 3) was used, generalizing to the scan protocol containing the 12 different acquisitions. A separate analysis was performed for the most severe metal artifacts in pellet R6 by means of two within-subject factors, notably reconstruction technique and O-MAR (“off,” “on”). Greenhouse–Geisser-produced* p* values were interpreted and a two-sided alpha of 5% was used as a significance level.

## Results

### No artifacts

#### CT number accuracy and noise values

**Fig. 3:**
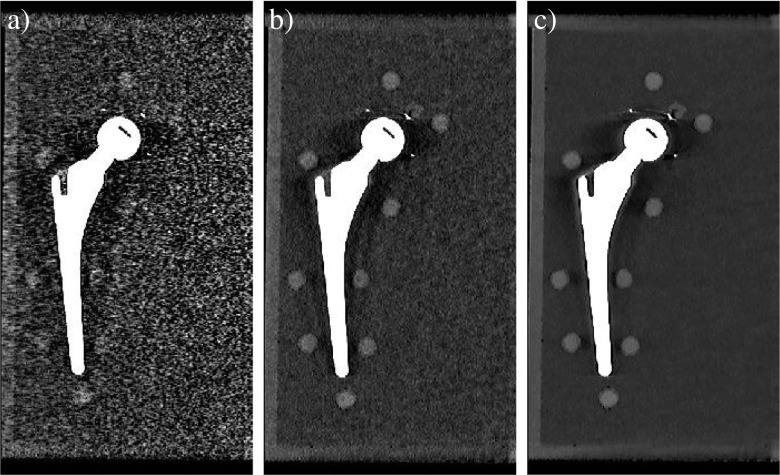
Images acquired at 140 kVp and 4.0 mGy reconstructed with** a** filtered back projection (FBP),** b** iDose^4^ level 4, and** c** IMR level 2 with the use of O-MAR. Lower noise values and improved overall image quality can be observed in images reconstructed with IMR level 2 compared with FBP and iDose^4^

Computed tomography numbers of the unaffected pellets, L0, L4, R0, and R4, were significantly lower for 140-kVp acquisitions compared with 120-kVp acquisitions (*p* < 0.001) and CT numbers in IMR reconstructions were systematically lower compared with iDose^4^ and FBP reconstructions (*p* < 0.005). Noise values or standard deviations were higher for FBP reconstructions at all dose levels and both kVp values compared with iDose^4^ and IMR reconstructions. In low-dose acquisitions in particular, noise values increased with FBP compared with iDose^4^ and IMR (Fig. 3). Noise values were lowest for 24.0 and 32.0 mGy for 120-kVp and 140-kVp results in all reconstructions respectively. With IMR, noise values were lowest compared with FBP and iDose^4^ reconstructions (*p* < 0.01) and CT numbers remained constant from high- to low-dose (Table 1).

**Table 1:** Reference CT numbers, signal-to-noise-ratios (SNRs), and contrast-to-noise-ratios (CNRs) for filtered back-projection (FBP), iterative reconstruction iDose^4^ (levels 2, 4, and 6), and iterative model-based reconstruction (IMR; levels 1, 2, and 3) at CT dose indexes (CTDI_vol_) of 40.0, 32.0, 24.0, 16.0, 8.0, and 4.0 mGy at 120 and 140- kVp without the influence of metal artifacts. Reference values were determined by averaging values of the unaffected pellets L0, L4, R0, and R4

	CTDI_vol_ (mGy)	FBP	iDose^4^ L2	iDose^4^ L4	iDose^4^ L6	IMR L1	IMR L2	IMR L3
CT numbers								
120- kVp	40.0	285 ± 37	283 ± 30	283 ± 25	283 ± 20	263 ± 15	263 ± 11	263 ± 6
	32.0	285 ± 43	284 ± 34	284 ± 29	283 ± 22	265 ± 16	265 ± 11	265 ± 7
	24.0	282 ± 41	284 ± 30	284 ± 25	283 ± 20	267 ± 14	268 ± 9	269 ± 5
	16.0	278 ± 60	279 ± 42	280 ± 35	281 ± 28	265 ± 17	266 ± 12	267 ± 6
	8.0	286 ± 79	284 ± 42	284 ± 36	282 ± 28	269 ± 17	269 ± 11	269 ± 5
	4.0	261 ± 114	282 ± 56	283 ± 48	282 ± 38	267 ± 20	268 ± 13	269 ± 6
140- kVp	40.0	259 ± 38	259 ± 30	259 ± 26	259 ± 20	243 ± 15	244 ± 11	244 ± 6
	32.0	264 ± 33	263 ± 26	263 ± 22	262 ± 17	245 ± 12	245 ± 8	246 ± 5
	24.0	254 ± 43	255 ± 33	255 ± 28	256 ± 22	241 ± 15	242 ± 10	243 ± 6
	16.0	260 ± 57	261 ± 37	261 ± 32	261 ± 25	247 ± 15	247 ± 10	247 ± 5
	8.0	244 ± 82	254 ± 42	255 ± 36	255 ± 27	246 ± 16	247 ± 11	248 ± 6
	4.0	258 ± 118	265 ± 56	264 ± 47	261 ± 37	247 ± 20	247 ± 13	247 ± 6
SNRs								
120- kVp	40.0	7.8 ± 1.1	9.7 ± 1.4	11.5 ± 1.7	14.7 ± 2.4	17.6 ± 2.1	25.3 ± 3.3	46.9 ± 14.8
	32.0	6.6 ± 0.8	8.5 ± 0.9	9.9 ± 1.0	12.8 ± 1.3	17.0 ± 1.5	24.1 ± 2.0	40.3 ± 4.2
	24.0	7.0 ± 0.9	9.6 ± 1.4	11.4 ± 1.6	14.4 ± 2.2	20.1 ± 3.7	29.9 ± 5.7	53.1 ± 13.0
	16.0	4.7 ± 0.6	6.7 ± 0.5	7.9 ± 0.5	10.2 ± 0.8	15.2 ± 1.0	22.9 ± 2.2	45.4 ± 5.6
	8.0	3.6 ± 0.2	6.7 ± 0.4	8.0 ± 0.5	10.2 ± 0.5	15.9 ± 1.5	24.7 ± 2.1	53.4 ± 8.2
	4.0	2.3 ± 0.5	5.1 ± 0.8	6.0 ± 0.9	7.5 ± 1.1	13.7 ± 0.8	21.3 ± 1.6	44.1 ± 9.3
140-kVp	40.0	7.1 ± 1.3	8.8 ± 1.6	10.4 ± 1.8	13.3 ± 2.4	16.4 ± 1.8	23.3 ± 2.8	41.5 ± 5.8
	32.0	8.1 ± 0.6	10.3 ± 1.0	12.3 ± 1.2	15.2 ± 1.7	20.7 ± 2.1	29.2 ± 2.0	50.5 ± 2.3
	24.0	5.9 ± 0.5	7.8 ± 0.6	9.2 ± 0.5	11.6 ± 0.5	15.9 ± 0.9	23.3 ± 0.8	40.3 ± 2.6
	16.0	4.6 ± 0.4	7.0 ± 0.5	8.3 ± 0.6	10.5 ± 0.7	16.9 ± 0.8	24.7 ± 1.7	47.2 ± 9.0
	8.0	3.1 ± 0.8	6.1 ± 0.7	7.2 ± 0.8	9.4 ± 1.2	15.0 ± 0.9	22.4 ± 0.5	45.0 ± 12.0
	4.0	2.2 ± 0.4	4.8 ± 0.7	5.6 ± 0.8	7.0 ± 1.0	12.4 ± 1.6	19.2 ± 2.4	39.7 ± 6.2
CNRs								
120- kVp	40.0	7.9 ± 0.6	9.8 ± 0.9	11.5 ± 1.0	14.1 ± 1.4	17.7 ± 1.1	23.1 ± 1.4	32.7 ± 1.9
	32.0	7.3 ± 0.8	9.3 ± 0.8	10.9 ± 0.9	13.7 ± 1.0	18.1 ± 1.4	24.5 ± 1.8	35.9 ± 1.9
	24.0	6.0 ± 0.5	8.1 ± 0.8	9.6 ± 1.0	12.1 ± 1.5	16.4 ± 1.5	23.1 ± 2.3	38.0 ± 3.8
	16.0	5.3 ± 0.3	7.8 ± 0.3	9.2 ± 0.4	11.6 ± 0.8	17.4 ± 1.3	25.4 ± 2.7	40.0 ± 7.2
	8.0	3.3 ± 0.7	6.0 ± 0.8	7.1 ± 1.0	8.8 ± 1.0	14.8 ± 1.6	22.2 ± 2.8	41.2 ± 6.6
	4.0	2.1 ± 0.7	5.0 ± 0.6	6.0 ± 0.7	7.6 ± 0.8	13.6 ± 0.5	21.3 ± 0.9	43.3 ± 5.7
140- kVp	40.0	7.7 ± 0.5	9.5 ± 0.5	11.1 ± 0.6	14.1 ± 1.2	17.3 ± 1.6	23.4 ± 2.9	34.9 ± 6.3
	32.0	6.8 ± 1.0	8.5 ± 1.1	9.9 ± 1.3	12.4 ± 1.8	15.9 ± 1.2	21.8 ± 1.8	33.0 ± 3.3
	24.0	6.1 ± 1.0	8.0 ± 1.3	9.4 ± 1.6	11.9 ± 2.1	16.2 ± 3.3	22.6 ± 5.4	35.1 ± 11.7
	16.0	4.8 ± 0.6	7.0 ± 0.6	8.3 ± 0.8	10.6 ± 0.9	16.7 ± 0.4	23.8 ± 0.8	38.7 ± 1.5
	8.0	3.2 ± 0.7	5.6 ± 0.7	6.6 ± 0.9	8.4 ± 1.0	14.8 ± 1.4	22.0 ± 2.7	38.2 ± 7.3
	4.0	2.3 ± 0.5	5.3 ± 0.8	6.2 ± 0.9	7.7 ± 1.1	13.7 ± 2.1	21.0 ± 2.9	40.8 ± 5.8

#### SNRs

In general, SNRs decreased from high- to low-dose for all reconstruction techniques and both kVp values. SNRs were higher in all acquisitions using IMR compared with iDose^4^ and FBP (*p* < 0.001). With IMR, peak SNRs were found at CTDI_vol_ of 24.0 for 120-kVp results. For 140-kVp results, peak SNRs were found at CTDI_vol_ of 32.0 mGy for all reconstruction techniques. These peak SNRs were caused by lower noise values at these dose levels, as CT numbers were constant for all reconstructions using iDose^4^ and IMR (Table 1).

#### CNRs

Contrast to noise ratios decreased from high- to low-dose for all reconstruction techniques and kVps except for IMR level 3 reconstructions. In IMR level 3 reconstructions, CNRs increased when decreasing the tube current, i.e., decreasing radiation dose (Table 1). CNRs in IMR reconstructions were higher compared with iDose^4^ and FBP and CNRs with iDose^4^ were higher compared with FBP (*p* < 0.001). Focusing on levels of noise reduction regarding iDose^4^ levels 2, 4, and 6 and IMR level 1, 2, and 3, higher levels of reconstruction level resulted in higher SNRs and CNRs owing to lower noise levels (Table 1).

When observing 120- and 140-kVp results for all dose levels, IMR results in noise reduction of more than 59% and SNRs and CNRs were more than a factor 2.3 and 2.2 higher in the case of IMR level 1, and there was a noise reduction of more than 83% and SNRs and CNRs were more than a factor 5.9 and 4.5 higher in the case of IMR level 3 compared with FBP reconstructions. At the low dose of 4.0 mGy, IMR levels 1, 2, and 3 showed 83%, 89%, and 95% lower noise values, a factor 6.0, 9.2, and 17.9 higher SNRs, and 5.7, 8.8, and 18.2 higher CNRs respectively, compared with standard FBP reconstructions, while maintaining constant CT numbers.

### Metal artifacts without O-MAR

As our main focus was to investigate dose reduction capabilities in the CT imaging of a metal hip prosthesis using IMR, we only focused on the pellet most affected by metal artifact, which was pellet R6 (Fig. 2). In pellet R6 metal artifacts were most pronounced, which was reflected by relatively large deviations of CT numbers, noise values, SNRs, and CNRs from unaffected reference values obtained from pellets L0, L4, R0, and R4.

Computed tomography numbers of pellet R6 were lower compared with unaffected pellets for all reconstruction techniques and acquisitions owing to the influence of metal. At a reduced radiation dose, CT numbers were clearly more deviated compared with reference values than in higher radiation dose acquisitions (Fig. 5). Largest deviations were observed in FBP reconstructions compared with iDose^4^ and IMR where IMR results showed the least deviations in CT numbers. Noise values or standard deviations of pellet R6 were increased and SNRs and CNRs were decreased compared with reference values because of the influence of metal.

### The combined use of IMR and O-MAR

**Fig. 4:**
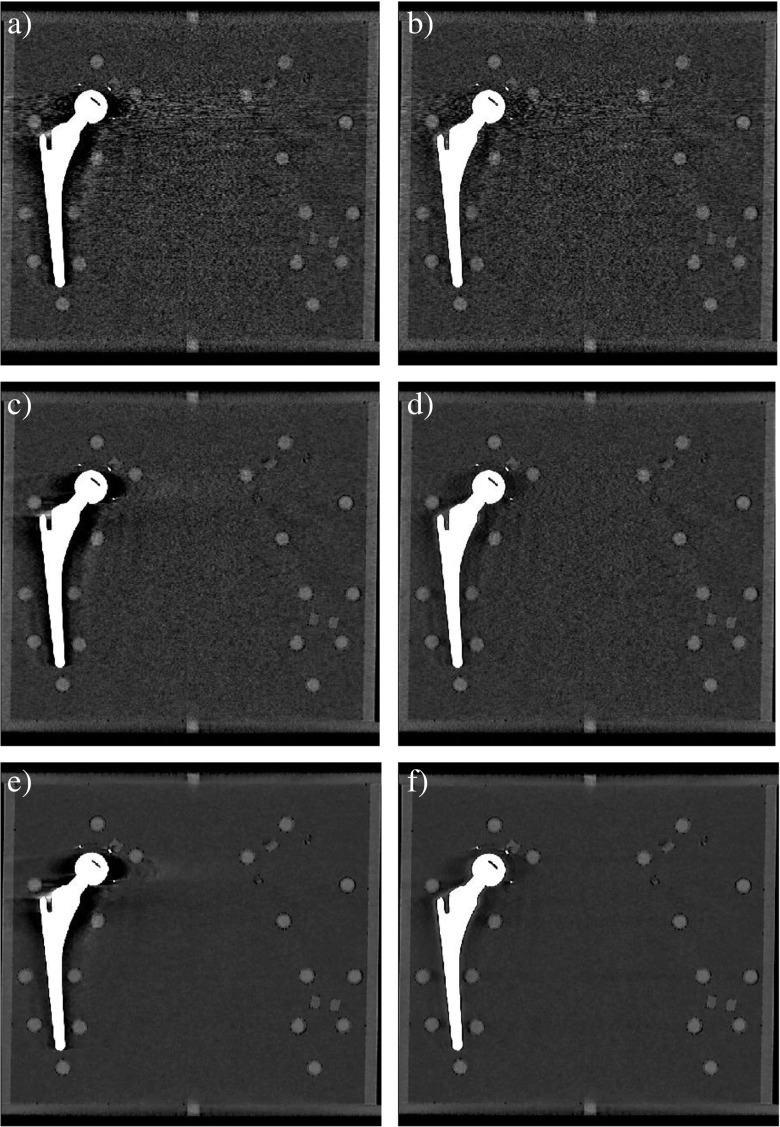
Images acquired at 140 -kVp and CDTI_vol_ of 24.0 mGy reconstructed with** a** FBP,** b** FBP + O-MAR,** c** iDose^4^ level 4,** d** iDose^4^ level 4 + O-MAR,** e** IMR level 2, and** f** IMR level 2 + O-MAR. IMR level 2 and O-MAR results (**f**) show the least noise with reduced metal artifacts compared with conventional FBP and iDose^4^ reconstructions

**Fig. 5:**
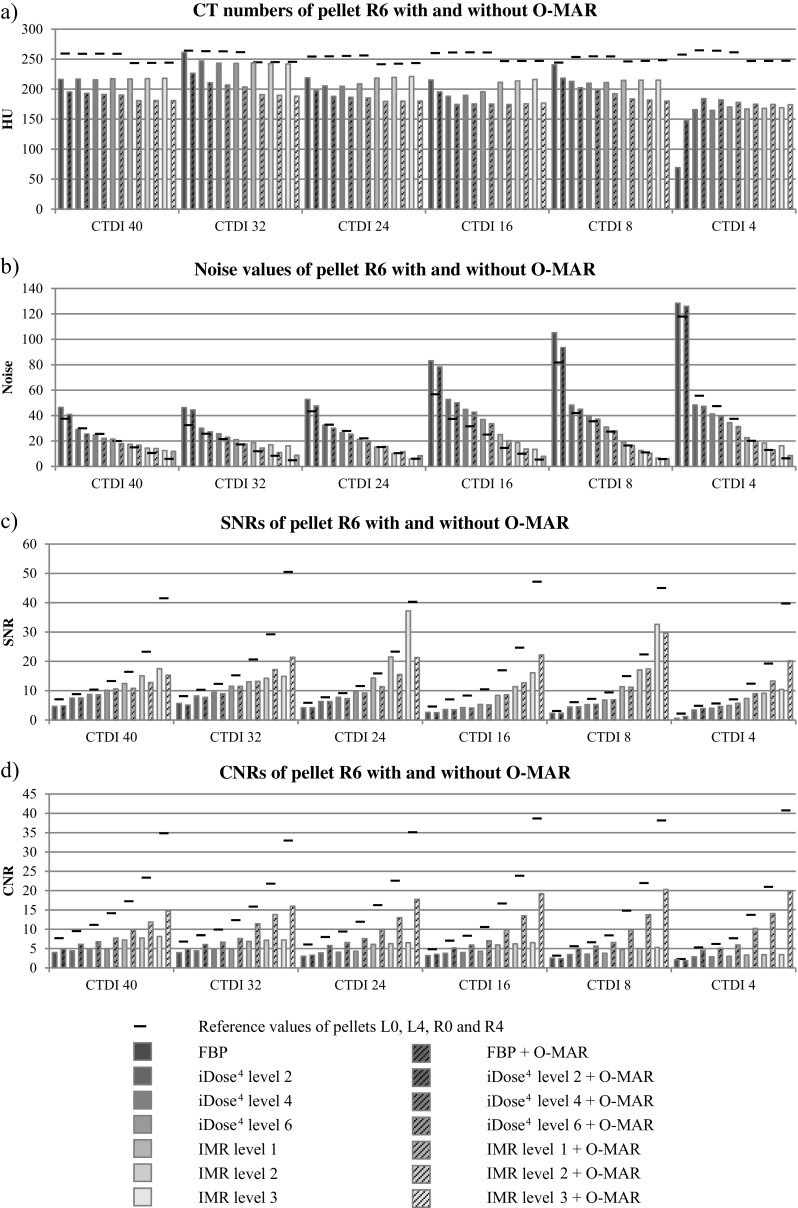
**a** CT numbers,** b** noise values,** c** SNRs, and** d** CNRs of pellet R6 with and without the use of O-MAR compared with reference values for all reconstructions and 140-kVp acquisitions from high- to low-dose

In general, O-MAR reduces metal artifacts in pellet R6 by improving SNRs (*p* > 0.01) and CNRs (*p* > 0.001) while decreasing noise values (*p* > 0.001; Figs. 4, 5). The use of O-MAR did not result in significant improvement of HU deviations in all acquisitions. Only in low-dose acquisitions did the use of O-MAR result in a correction of HUs deviated by metal artifact toward reference values of unaffected pellets (Fig. 5a). CT numbers in IMR and O-MAR reconstructions were constant from high- to low-dose.

**Fig. 6:**
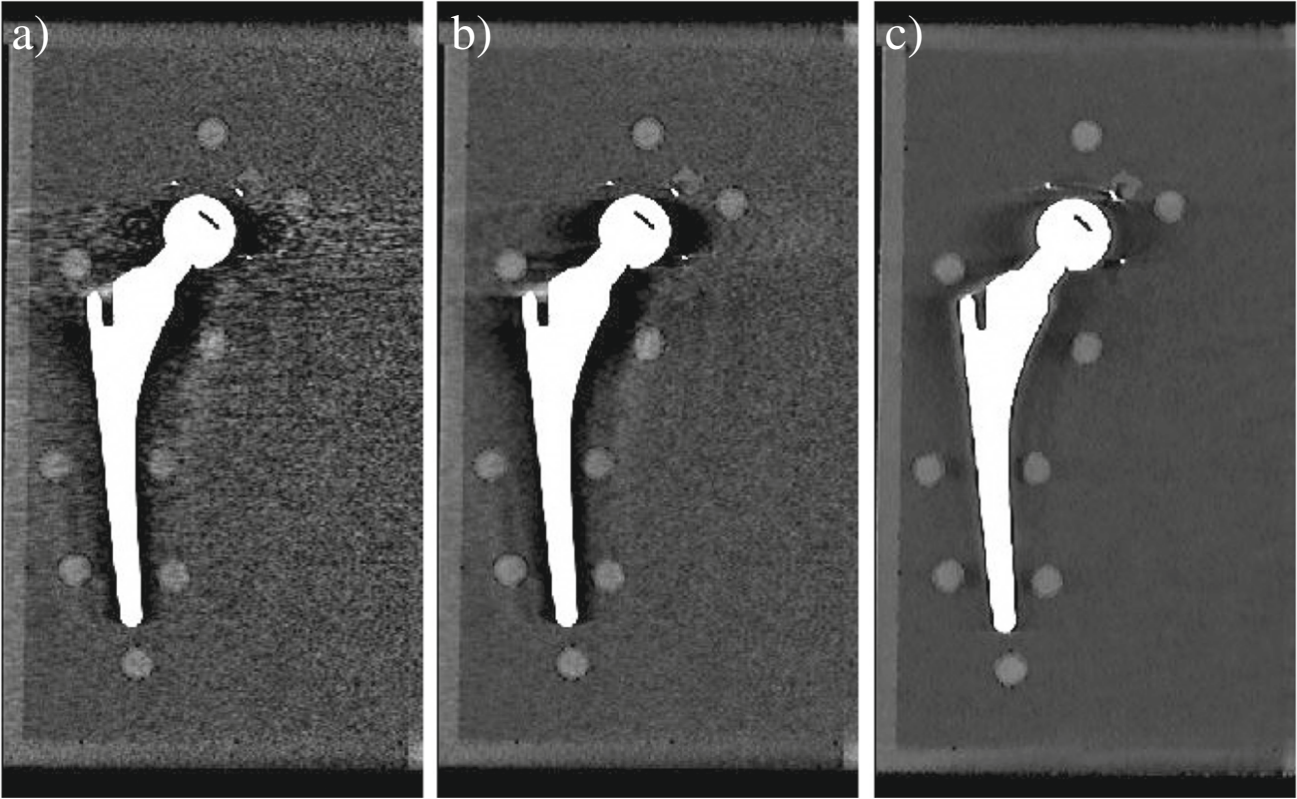
A 24.0-24.0 mGy (instead of 24.0-mGy)mGy acquisition at 140 - kVp reconstructed with **a** FBP and **b** iDose^4^ level 4.** c** A 4.0-4.0 mGy (instead of 4.0-mGy)mGy acquisition at 140- kVp reconstructed with IMR level 2 and O-MAR. IMR and O-MAR results in** c** show a clearly improved image quality with reduced metal artifacts compared with** a** and** b** reconstructed with FBP and iDose^4^, while reducing radiation dose by 83%

O-MAR decreased noise values of pellet R6 when combined with FBP, iDose^4^ and IMR in nearly all acquisitions. Greatest noise reduction was observed at low-dose using IMR levels 1, 2, and 3. Also, deviations in SNRs and CNRs of pellet R6 compared with reference values were largest in IMR reconstructions owing to the clearly higher reference values (Fig. 5). SNRs were not improved by O-MAR in all acquisitions. Absolute SNR improvements by O-MAR were largest at the low-dose acquisition of 4.0 mGy for all reconstructions techniques and were more than a factor 2 higher when combined with IMR compared with FBP and iDose^4^ in 4.0 mGy acquisitions. With the use of O-MAR, CNRs were strongly improved where largest improvements were observed when combined with IMR. Fig. 6a illustrates that a 4.0-mGy acquisition at 140 kVp reconstructed with IMR and O-MAR shows superior image quality compared with 24.0-mGy acquisitions at 140- kVp reconstructed with FBP (Fig. 6a) and iDose^4^ level 4 (Fig. 6b), which corresponds to a radiation dose reduction of 83% with reduced metal artifacts.

## Discussion

This phantom study shows that the iterative model-based reconstruction technique, IMR, improves overall image quality with higher SNRs (*p* < 0.001) and CNRs (*p* < 0.001) and lower noise values (*p* < 0.01) compared with FBP and iDose^4^ while maintaining constant CT numbers from high- to low-dose. In the case of metal artifacts, 140-kVp acquisitions are advised due to smaller deviations in CT numbers, noise values, SNRs, and CNRs compared with reference values than in 120-kVp acquisitions. The lower CT numbers for IMR results compared with FBP and iDose^4^ results are in concordance with previous work and can be explained using a different reconstruction filter. The IMR reconstruction filter uses edge enhancement, which can influence CT numbers in small objects [8]. The orthopedic metal artifact reduction algorithm O-MAR reduced metal artifacts by improving SNRs (*p* < 0.01) and CNRs (*p* < 0.01) while decreasing noise values (*p* < 0.01), and showed the largest absolute improvements in low-dose acquisitions where metal artifacts were most pronounced. O-MAR is most effective when combined with IMR based on the largest CNR improvements. Subsequently, O-MAR is most effective with an increased reconstruction level for both iDose^4^ and IMR. Regarding deviated CT numbers due to the influence of metal artifacts, O-MAR only improved CT number accuracy in low-dose acquisitions with the most severe artifacts. In general, larger deviations compared with reference values due to the influence of metal artifacts result in larger absolute corrections by O-MAR of these deviations. However, reference values of unaffected pellets were not reached.

Our results showed that 4.0-mGy (17%) 140-kVp acquisitions reconstructed with IMR and O-MAR resulted in comparable or higher SNRs and CNRs and lower noise values compared with 24.0-mGy (100%) acquisitions reconstructed with FBP or iDose^4^ with O-MAR with constant CT numbers. This enables a radiation dose reduction of 83% based on this quantitative phantom study. There are no data available on dose reduction capabilities in the CT imaging of metal implants using iterative model-based reconstruction without or with the use of metal artifact reduction software. However, our results are in concordance with those of several recent studies, which show that model-based iterative reconstruction techniques are able to reduce image noise up to 75–88% and radiation dose up to 75–92% and improve SNRs and CNRs in other CT protocols too [9–16]. In a previous study, we showed that IMR improves overall image quality and that O-MAR is most effective in severe artifacts and when combined with IMR in improving CT number accuracy, SNRs, and CNRs, while decreasing noise [8].

This study mainly focused on improving image quality and reducing metal artifacts using IMR and O-MAR at regular dose levels instead of focusing on dose-reduction capabilities. However, it needs to be stated that the titanium–aluminum–vanadium prosthesis used in the current study, and most often used in our patient population, resulted in less severe artifacts compared with the MoM prosthesis used in our previous study, which was composed of a cobalt–chrome–molybdenum alloy with a greater atomic weight.

O-MAR did not reduce differences in the CT numbers of pellet R6 compared with reference values in all acquisitions, but mainly in low-dose acquisitions (Fig. 5). This confirms earlier findings stating that O-MAR is most effective in severe artifacts, because at low-dose acquisitions the reduced number of photons induces more severe artifacts. A recent study by Boudabbous et al. showed that model-based iterative reconstruction reduces the size of metal artifacts on CT images and allows a better analysis of the soft tissue surrounding the metal implant compared with FBP [22]. To our knowledge, this is the only study investigating metal artifacts using MBIR; however, without the use of metal artifact reduction software and without investigating dose reduction capabilities. We observed no differences in metal artifacts in IMR and FBP results, as artifacts did not seem to differ in size or severity (Fig. 4a, c, e).

In general, noise increases when lowering CT radiation dose. In our results, 24.0 mGy and 32.0 mGy showed lowest noise values for 120-kVp and 140-kVp results respectively. As we only made a single scan for each condition, there may be some uncertainty in the estimated noise levels that might be larger than the differences found between those conditions. This is more likely for the cases where the noise levels are low and differences in noise levels are relatively small than for the IMR results. In regions affected and unaffected by metal and with and without the use of O-MAR, overall image quality is superior using IMR levels 1, 2, and 3 compared with FBP and iDose^4^ levels 2, 4, and 6. Subsequently, image quality in IMR level 3 results, with the highest level of noise reduction, is superior to that of IMR level 1 and 2 results. CNRs of unaffected pellets in images reconstructed with IMR level 3 stood out, because a decrease in radiation dose led to an increase in CNRs. A possible explanation for the observed IMR trends could be its (over-)effectiveness in noise reduction. CNRs increased because of a decrease in noise levels in the background ROIs, where CT numbers showed a slight increase from high- to low-dose acquisitions. As noise increases at a decreased radiation dose, noise is highest at a CTDI_vol_ of 4.0 mGy. IMR level 3 is best capable of dealing with these increased noise levels. A side effect of this magnitude of noise reduction in low-dose images is the increasing smoothing effect. McCollough et al. found in a phantom study that for radiation dose reductions of more than 25%, the ability to resolve 6-mm rods in the ACR CT accreditation phantom can be lost [23]. When detecting soft-tissue pathological conditions in THA patients involving low-contrast lesions, relatively low noise levels and high spatial resolution are required. Our results showed that noise levels also remain low using IMR in low-dose acquisitions, thereby probably enabling a dose reduction in clinical practice too. However, as stated before, caution should be taken in the case of such dose reduction steps as the smoothing effect could lead to a loss of small detail and low-contrast detectability. Den Harder et al. recently found that the use of iDose^4^ did result in an increased number of false-positive findings in the computer-aided detection of pulmonary nodules at reduced dose levels and that CT volume measurements of pulmonary nodules at a low dose using IMR were lower compared with iDose^4^ and FBP [24, 25]. Kaasalainen et al. [16] evaluated image noise, soft tissue contrast and bone tissue contrast in a study using pediatric anthropomorphic phantoms, while reducing radiation dose in craniosynostosis CT. They found that while reducing radiation dose by up to 83% and 88%, image quality remained adequate. Besides the high bone tissue contrast, as we investigated in our study, soft tissue contrast remained more or less constant while reducing radiation dose using MBIR. Furthermore, a study by Brænne et al. showed that iterative algorithms, specifically model-based iterative reconstruction algorithms, improve lesion detectability of low-contrast lesions in a liver phantom; however, this may result in poorer image quality when applying aggressive radiation dose reduction [26]. Results of these studies involving radiation dose reduction using (model-based) iterative reconstruction methods all state that caution should be taken. Additionally, especially in low-dose situations, photon starvation artifacts will be more apparent owing to the reduced number of photons. Even though we did not observe signs of increased photon starvation artifacts, we are aware of the possible side-effects of dose reduction, especially in the case of severe metal artifacts and relatively large patient sizes in THA patients.

Our study has several limitations. We have only performed a quantitative analysis using a standardized measurement template. Additional subjective image quality scoring could provide more insights into the clinical usefulness and opinions of radiologists in evaluating low-dose CT scans. Second, the hydroxyapatite/calcium carbonate pellets with a high density resulted in high contrast values between the pellets and their background. Adding pellets with different densities or soft-tissue structures can provide more insights into the possible additional clinical value in patients, especially regarding low-contrast detectability in low-dose situations. Furthermore, adding total hip arthroplasties consisting of different metal alloys can provide important information regarding the influence of different metal alloys while reducing the radiation dose, as heavier metals may impede radiation dose reduction. At last, known smoothing effects due to noise reduction could result in a loss of small objects or details, which needs to be investigated by subjective image quality scoring. Therefore, most important regarding future prospective, in order minimize radiation dose levels in the CT imaging of total hip arthroplasty in patients, a clinical patient study needs to be started with qualitative and quantitative image quality scoring focusing on the evaluation of the musculoskeletal anatomy and pathology.

We have used a total hip arthroplasty phantom reflecting the dimensions of a patient with an average BMI. We addressed image quality, metal artifacts, and the degree of MAR by quantifying CT numbers, noise, SNRs, and CNRs. We addressed noise as the standard deviation of pixel intensities within a ROI, and know that both noise and artifact influence the standard deviation. Based on previous studies, we conclude that the measured noise reduction by O-MAR is mainly caused by a reduction of metal artifacts resulting in a lower standard deviation, as O-MAR has no influence on images without metal artifacts.

Based on quantitative analysis on CT number accuracy, noise values, SNRs, and CNRs, we conclude that with the combined use of model-based iterative reconstruction (IMR) and orthopedic metal artifact reduction (O-MAR), image quality parameters are maintained at a reduction in radiation dose of 83% compared with FBP and iDose^4^ in the CT imaging of a total hip arthroplasty phantom. Although results of this phantom study are promising, future clinical studies are needed to determine if the results of this phantom study can lead to radiation dose reduction in THA patients.
